# Canonical Pathways Rewiring in Alzheimer’s Disease

**DOI:** 10.3390/ijms27114835

**Published:** 2026-05-27

**Authors:** Alejandro Pinta-Castro, Gabriela Michel-Ureña, Alejandra Paulina Pérez-González, Guillermo De Anda-Jáuregui, Enrique Hernández-Lemus

**Affiliations:** 1 Facultad Mexicana de Medicina, Universidad La Salle, México City 14100, Mexico; alejandro.pinta@lasallistas.org.mx (A.P.-C.);; 2 División de Genómica Computacional, Instituto Nacional de Medicina Genómica, México City 14610, Mexico; paulinapglz.99@gmail.com (A.P.P.-G.); gdeanda@inmegen.gob.mx (G.D.A.-J.); 3 Programa de Doctorado en Ciencias Biomédicas, Universidad Nacional Autónoma de México, México City 04510, Mexico; 4 Investigadores por México, Secretaría de Ciencia, Humanidades, Tecnología e Innovación (SECIHTI), Mexico City 03940, Mexico

**Keywords:** canonical pathways, Alzheimer’s disease, co-expression networks, GABAergic, glutamatergic, neurexins and neuroligins, SNARE, synaptogenesis

## Abstract

Alzheimer’s disease (AD) is a multifactorial neurodegenerative disorder characterized by the simultaneous disruption of interconnected molecular pathways, yet the structural mechanisms underlying this transcriptional disintegration remain poorly characterized. To address this, we constructed condition-specific gene co-expression networks from DLPFC bulk RNA-seq data, using a mutual-information (MI) framework with infomap community partitioning. Functional enrichment of network communities via Ingenuity Pathway Analysis (IPA) identified GABAergic signaling, SNARE complex assembly, Synaptogenesis, and neurexin and neuroligin interactions as significantly overrepresented pathways. Integration of node degree with condition-specific average expression revealed coordinated topological centralization of key synaptic genes—including NRXN2, LRRTM1, DLGAP3, and SHANK1—alongside a widespread transcriptional downregulation in GABAergic and Synaptogenesis modules. A shortest-path analysis revealed a consistent expansion of intra-pathway distances across all evaluated canonical pathways in AD, a pattern statistically consistent with reduced local co-expression cohesion. These findings reframe Late-Onset Alzheimer’s Disease (LOAD) as an active structural-rewiring process, in which the observed topological centralization pattern seems to be consistent with a consolidation of co-expression around synaptic components, though we cannot exclude that shifts in cellular composition contribute to this signal in bulk RNA-seq data.

## 1. Introduction

Alzheimer’s Disease (AD) is the main cause of dementia, accounting for up to 70% of all cases worldwide [1,2]. Late-Onset Alzheimer’s Disease (LOAD) is typically defined by symptom onset at or after 65 years of age and clinically presents as a progressive disorder characterized by early and prominent episodic memory impairment, which gradually extends to other cognitive domains, including language, visuospatial abilities, and executive function [3]. As a result, AD is rapidly becoming one of the most expensive, lethal, and burdensome diseases of this century [4]. The global prevalence of dementia was approximately 50 million people worldwide in 2018 and is projected to triple by 2050 [4,5]. Despite significant advances in understanding AD pathogenesis, current treatment strategies generally only ameliorate symptoms, and an effective cure remains elusive [6].

Since genes do not function in isolation, but rather as part of complex, interconnected modules, deciphering these widespread pathological alterations requires a holistic approach. In this regard, gene co-expression networks provide a systems biology framework for analyzing the concerted regulation of molecular entities and investigating the dynamic transcriptional landscape in disease [7,8].

This network-based approach allows for the identification of deregulation by characterizing structural changes and reorganizations across three distinct topological scales: macroscopic, microscopic, and mesoscopic. At the macroscopic (or global) scale, network analytics evaluate the overall architecture and connectivity patterns of the entire system, such as degree distributions and global cohesion. At the microscopic (or local) scale, this methodology facilitates the detection of individual node properties, such as highly interconnected “hub genes”, which play a central role in maintaining system stability and coordinating functional processes [9]. Moreover, it also allows for the characterization of mesoscopic alterations (the intermediate structural level), reflected in the reorganization of genes into distinct transcriptional modules or communities [10]. Although core biological functions may remain relatively preserved, the specific gene composition within these modules can undergo substantial rearrangement. In this context, functional enrichment analyses of restructured communities and high-betweenness centrality genes have consistently been associated with processes relevant to disease pathology, including synaptic function and synaptic vesicle dynamics [11].

Unlike traditional analyses that rely solely on canonical pathways, which are established representations of biological signaling cascades based on the prior literature, our data-driven approach captures the actual, condition-specific topological architecture of the transcriptome. By performing co-expression network analysis and integrating it with canonical pathway enrichment, we map these functional co-expression arrangements for each phenotype onto established biological knowledge. This combined strategy aims to interpret the dynamic rewiring of the brain, providing insights into the key molecular networks underlying LOAD pathogenesis. Therefore, the primary problem this study seeks to address is the incomplete understanding of how the brain’s transcriptional architecture structurally and functionally rewires in response to the disease, particularly within highly vulnerable regions like the dorsolateral prefrontal cortex (DLPFC).

Building upon the foundational network characterization of LOAD established by Pérez-González et al. [12], who described the global topological reorganization of gene co-expression in the DLPFC, the present study advances this framework by shifting the analytical focus from network-wide properties to pathway-level resolution. While the preceding work characterized macroscopic and mesoscopic alterations, including modularity changes, hub gene redistribution, and community rearrangements, the current analysis interrogates how these global topological shifts manifest within specific canonical synaptic pathways. By integrating condition-specific expression profiles with intra-pathway shortest-path distances and quadrant-based connectivity mapping, we provide a pathway-resolved characterization of transcriptional rewiring in AD that complements and extends the network-level findings of the prior study.

## 2. Results

Transcriptional changes in the DLPFC of AD subjects compared to neurologically healthy controls based on average expression analysis identified transcriptional dysregulation across the cortex, characterized predominantly by the downregulation of genes encoding key synaptic and metabolic components. Transcriptional suppression (FDR < 0.05) was observed in fundamental transport and vesicular machinery. For instance, the vesicle fusion regulator *STXBP1* was significantly downregulated (Log2FoldChange [L2FC] = −0.190; FDR = 0.0037). Similar significant expression deficits were detected for *STX1B* (L2FC = −0.182; FDR = 0.0075), *SYT3* (L2FC = −0.231; FDR = 0.017), and the cell adhesion molecule *LRRTM1* (L2FC = −0.246; FDR = 0.0028). Certain central regulatory kinases, such as *CDK5* (L2FC = −0.168; FDRj = 0.018), also exhibited marked downregulation in the AD cohort.

### 2.1. Network Inference and Modular Organization

The AD network exhibited a higher modularity strength (Q = 0.28) compared to the control network (Q = 0.20), indicating a more segregated community structure within the pathological state. The control graph was partitioned into 71 functional communities, whereas the AD graph was partitioned into 68 communities. Throughout this manuscript, the terms “module” and “functional community” refer to the same underlying structure but emphasize different aspects. A “module” denotes a group of genes defined by networking topology, specifically genes that are more densely connected to each other than to the rest of the network. The term “functional community” is used when referring to the same module after functional enrichment analysis, reflecting the biological interpretation that genes which co-express together tend to participate in shared biological processes. This dual terminology is consistent with established practice in gene co-expression network analysis [13].

**Figure 1:**
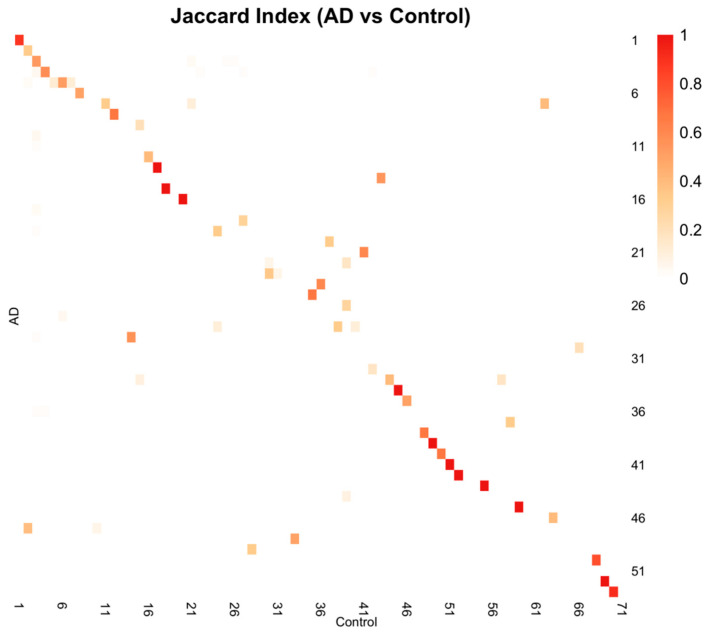
Jaccard index heatmap comparing gene membership similarity between communities in AD and Control networks. Each cell represents the Jaccard similarity between a pair of communities. The *Y*-axis shows the AD’s communities, while the *X*-axis represents the control’s communities.

Reorganization of gene communities was assessed quantitatively by comparing modular partitions of AD and control networks. Community similarity between the two networks was quantified using Normalized Mutual Information (NMI), an information-theoretic metric that measures the degree of correspondence between two partitions, ranging from 0 (completely different partitions) to 1 (identical partitions). An NMI score of 0.4479 indicated a rearrangement of gene memberships across modules. To further characterize the nature of the increased modularity observed in the AD network, we quantified the distribution of edges within and between communities. The AD network exhibited a higher proportion of inter-community edges (3.8%, *n* = 1058) compared to the control network (2.2%, *n* = 606), suggesting that co-expression becomes more compartmentalized under pathological conditions (see Table S4), while simultaneously maintaining a higher modularity strength (Q = 0.28 vs. Q = 0.20). Furthermore, the Jaccard similarity index was employed to evaluate the persistence of specific structural communities (Table S2). Global edge similarity across networks was around 68.39%. Module-level comparisons revealed a spectrum of conservation; while certain communities maintained high structural fidelity (e.g., community 1 exhibited a Jaccard index of 0.88), others displayed significant divergence (e.g., AD community 4 relative to control community 4 yielded a Jaccard index of 0.58, and AD community 5 mapped to control community 6 with an index of 0.51) (see Figure 1).

### 2.2. Pathway Enrichment

Following community partitioning, the structural communities were submitted to IPA [14] for functional characterization. IPA performed an overrepresentation analysis to identify biological functions disproportionately clustered within specific communities. Based on this approach, pathways associated with synaptic structure and function were identified as significantly overrepresented in the AD network communities. Specifically, “synapse organization” (GO:0050808) emerged as highly significant (FDR = 0.0046), driven by the coordinated mapping of genes including *NRXN2*, *LRRTM1*, *DLGAP3*, and *SHANK1*. Similarly, “synapse assembly” (GO:0007416) was identified as significantly overrepresented (FDR = 0.0074). These pathways were subsequently selected for detailed downstream evaluation based on their statistical significance and biological relevance to AD pathophysiology.

### 2.3. Network Repositioning Across Median-Defined Quadrants

**Figure 2:**
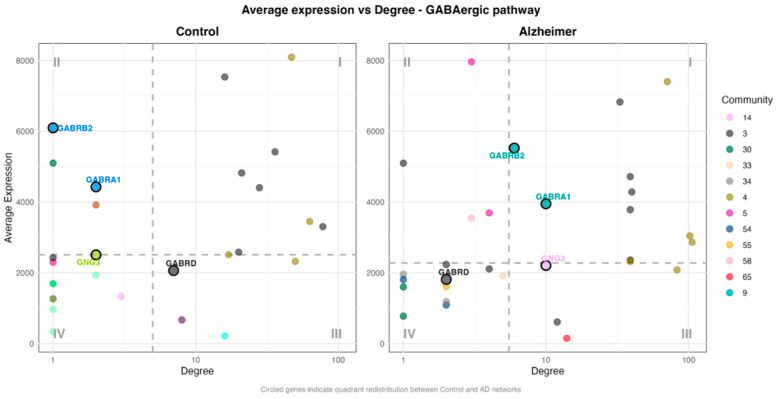
Average expression versus degree of interconnectivity in the GABAergic pathway. Dashed lines represent the median values for average expression (horizontal) and degree of interconnectivity (vertical), defining the quadrant boundaries. Node colors indicate the community membership of each gene within the condition-specific co-expression network and may differ between control and AD panels, reflecting changes in community assignment. Larger outlined nodes indicate genes that underwent quadrant redistribution between control and AD networks.

**Figure 3:**
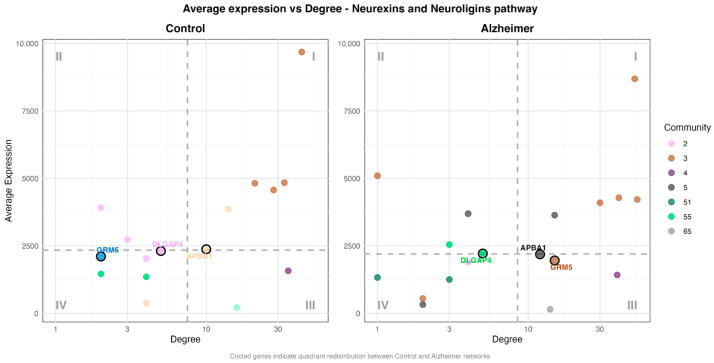
Average expression versus degree of interconnectivity in the neurexins and neuroligins pathway. Dashed lines represent the median values for average expression (horizontal) and degree of interconnectivity (vertical), defining the quadrant boundaries. Node colors indicate the community membership of each gene within the condition-specific co-expression network and may differ between control and AD panels, reflecting changes in community assignment. Larger outlined nodes indicate genes that underwent quadrant redistribution between control and AD networks.

**Figure 4:**
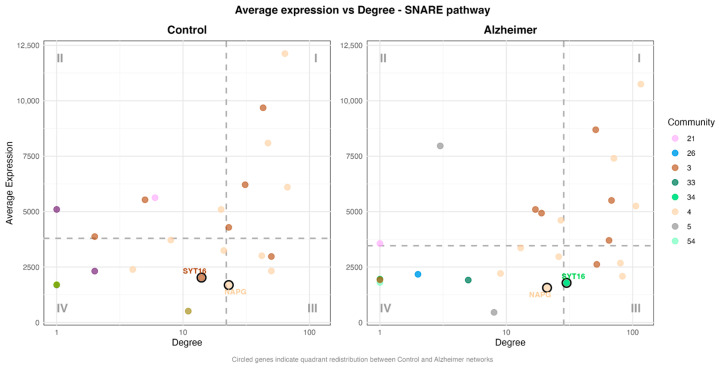
Average expression and interconnectivity in SNARE pathways. Dashed lines represent the median values for average expression (horizontal) and degree of interconnectivity (vertical), defining the quadrant boundaries. Node colors indicate the community membership of each gene within the condition-specific co-expression network and may differ between control and AD panels, reflecting changes in community assignment. Larger outlined nodes indicate genes that underwent quadrant redistribution between control and AD networks.

**Figure 5:**
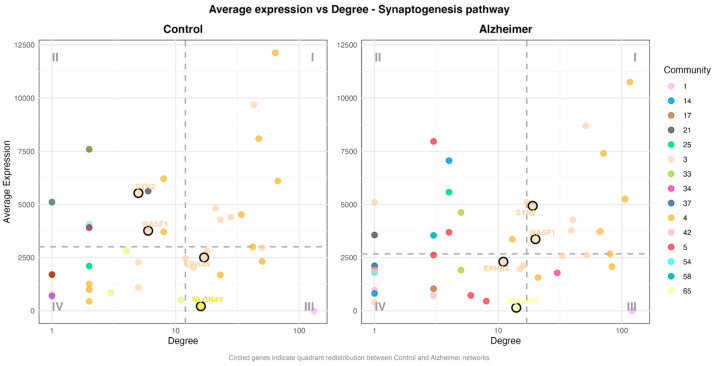
Average expression versus degree of interconnectivity in the Synaptogenesis pathway. Dashed lines represent the median values for average expression (horizontal) and degree of interconnectivity (vertical), defining the quadrant boundaries. Node colors indicate the community membership of each gene within the condition-specific co-expression network and may differ between control and AD panels, reflecting changes in community assignment. Larger outlined nodes indicate genes that underwent quadrant redistribution between control and AD networks.

The relationship between transcriptional magnitude and network connectivity was evaluated by mapping genes onto a two-dimensional expression–degree space, where degree refers to the number of co-expression edges connecting a given gene to other genes within the network, serving as a proxy for the gene’s topological influence within the transcriptional program. Quadrants were defined using the median expression and median degree values across the pathway-specific gene set as threshold boundaries (dashed lines; Figure 2, Figure 3, Figure 4 and Figure 5). The upper-right quadrant (Quadrant I) comprises genes with both above-median average expression and above-median connectivity degree, representing highly expressed and highly interconnected nodes. The upper-left quadrant (Quadrant II) included genes with above-median expression but below-median connectivity. The lower-right quadrant (Quadrant IV) corresponded to genes with above-median connectivity but below-median expression, while the lower-left quadrant (Quadrant III) contained genes with both below-median expression and low connectivity. This framework enabled the systematic characterization of coordinated transcriptional and topological shifts between the control and AD network (Table 1).

Across all four canonical pathways, a consistent pattern of dissociation between transcriptional output and topological centrality was observed in the AD network. In the GABAergic pathway, 54.5% of genes showed an increased degree in AD while 69.7% exhibited decreased average expression, with 12.1% of genes (*n* = 4 out of 33) undergoing quadrant redistribution. In the neurexins and neuroligins pathway, 50% of genes gained connectivity despite 88.9% showing reduced expression, with 16.7% of genes (*n* = 3 out of 18) shifting quadrants. The SNARE pathway showed the most pronounced connectivity gain, with 80% of genes increasing their degree while 100% exhibited decreased expression, and 10% of genes (*n* = 2 out of 20) changing quadrants. Finally, the Synaptogenesis pathway showed 68.6% of genes gaining connectivity alongside a widespread reduction in expression (97.1%), with 11.4% of genes (*n* = 4 out of 35) undergoing quadrant redistribution (Table S3).

#### 2.3.1. GABAergic Pathway

Within the GABAergic pathway, *GABRB2* and *GABRA1* underwent quadrant redistribution from Quadrant II (high expression, low connectivity) to Quadrant I (high expression, high connectivity), driven by a marked increase in interconnectivity. In contrast, *GABRD* shifted from Quadrant III to Quadrant IV, reflecting a loss of topological centrality while maintaining below-median expression. Finally, *GNG3* shifted from Quadrant IV to Quadrant III, reflecting a gain in relative connectivity while maintaining below-median average expression, consistent with the topological centralization pattern observed across the GABAergic pathway (see Figure 2).

#### 2.3.2. Neurexins and Neuroligins Pathway

Within the neurexins and neuroligins pathway, three genes underwent quadrant redistribution in the AD network. *APBA1* shifted from Quadrant I to Quadrant III, reflecting a reduction in transcriptional output while maintaining above-median connectivity. *DLGAP4* shifted from Quadrant IV to Quadrant II, reflecting a gain in transcriptional output while maintaining below-median connectivity. Finally, *GRM5* shifted from Quadrant IV to Quadrant III, reflecting a gain in connectivity while maintaining below-median expression (see Figure 3).

#### 2.3.3. SNARE Pathway

Within the SNARE pathway, *NAPG* shifted from Quadrant III to Quadrant IV, reflecting a loss of topological centrality while maintaining below-median expression. In contrast, SYT16 shifted from Quadrant IV to Quadrant III, reflecting a gain in connectivity while maintaining below-median expression (see Figure 4).

#### 2.3.4. Synaptogenesis Pathway

Within the Synaptogenesis pathway, four genes underwent quadrant redistribution. In the AD network, SYN2 and WASF1 shifted from Quadrant II to Quadrant I, reflecting a coordinated gain in topological centrality while maintaining above-median expression. In contrast, EPHA4 and NLGN4Y shifted from Quadrant III to Quadrant IV, reflecting a loss of connectivity while maintaining below-median expression (see Figure 5).

#### 2.3.5. Condition-Specific Enrichment Patterns

Notably, certain pathways displayed condition specific enrichment patterns. The GABA receptor pathway was significantly enriched only in the control network, whereas the Glutamatergic pathway was exclusively enriched in the AD network. This asymmetrical enrichment highlights differences in pathway representation between conditions.

### 2.4. Shortest-Path Distances in Modules

**Figure 6:**
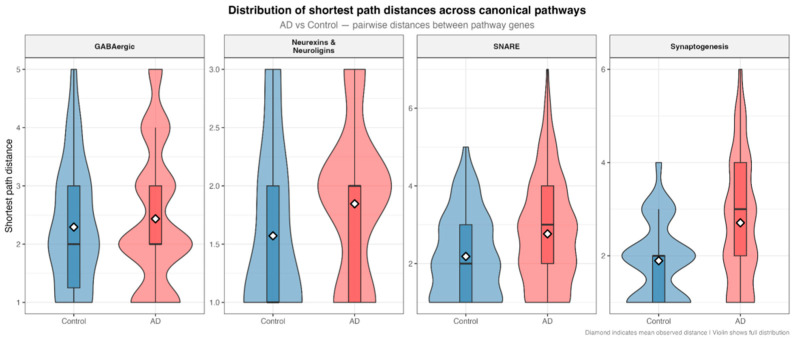
Distribution of pairwise shortest-shortest path distances across canonical pathways in AD and Control co-expression networks. Each violin represents the full distribution of distances between all gene pairs within the pathway. Box plots show interquartile range and median. The diamond indicates the mean observed distance. Red: AD network; Blue: Control Network.

To evaluate the local co-expression cohesion and structural integrity of the highly affected modules, shortest-path distances were computed between gene nodes within the specific canonical pathways already described. Across all evaluated pathways, the AD network exhibited a distinct increase in mean shortest distances compared to the control network, reflecting a loosening of topological coupling. The expansion of these path lengths, alongside the emergence of fully disconnected gene pairs in the pathological state, quantitatively demonstrates that the transcriptional rewiring in LOAD yields a less communicative architecture (see Figure 6; Figure S1).

**Figure 7:**
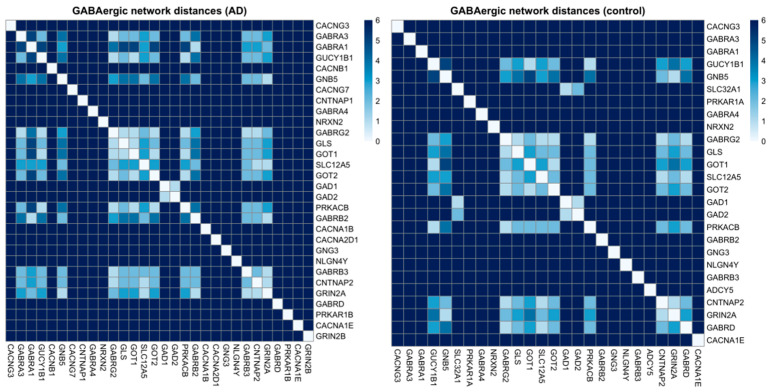
Heatmaps of shortest-path distances in the GABAergic network comparing AD and control samples.

**Figure 8:**
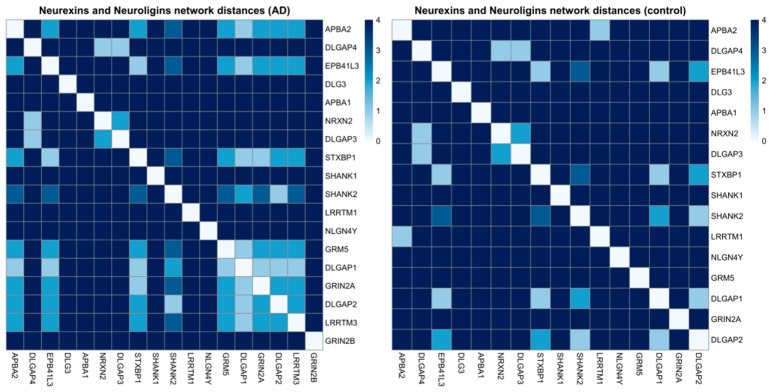
Heatmaps of shortest-path distances in the neuroligins and neurexins network comparing AD and control samples.

**Figure 9:**
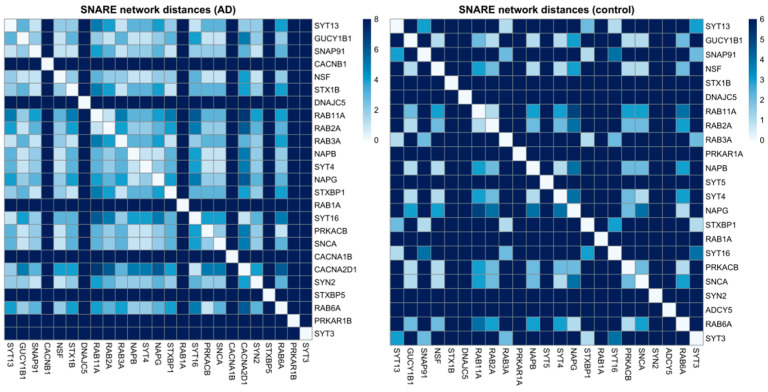
Heatmaps of shortest-path distances in the SNARE network comparing AD and control samples.

**Figure 10:**
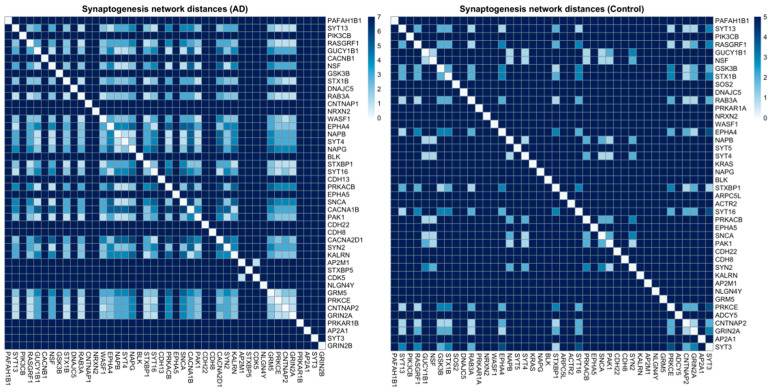
Heatmaps of shortest-path distances in the Synaptogenesis network comparing AD and control samples.

The GABAergic signaling pathway exhibited an increase in mean shortest-path distance from 2.293 in the control network to 2.435 in the AD network (Z = 4.56, *p* = 0.002; see Figure 6 and Figure 7). The neurexins and neuroligins network, which maintained the tightest topological coupling overall, also suffered a loss of efficiency, increasing the adjacent mean distance of 1.571 in the control network to 1.846 in the AD network (Z = 2.19, *p* = 0.029; see Figure 6 and Figure 8). The loss of co-expression cohesion was most pronounced within the SNARE and Synaptogenesis pathways. The SNARE pathway experienced a substantial topological expansion, with its mean internal distances increasing from 2.183 in control to 2.765 in the AD network (Z = 6.18, *p* < 0.001; see Figure 6 and Figure 9). Finally, the Synaptogenesis pathway displayed a mean shortest distance of 1.894, which expanded to 2.708 in the AD network (Z = 5.17, *p* = 0.004; see Figure 6 and Figure 10). Full permutations test results for all pathways are provided in Table S1. A summary of mean shortest-path distances across all canonical pathways is presented in Table 2.

## 3. Discussion

### 3.1. Confirming the Pathological Baseline

Following our functional analysis, synaptic-related pathways, specifically GABAergic signaling, glutamatergic signaling, SNARE complex, Neurexins and Neuroligins interactions, and Synaptogenesis, emerged as significantly overrepresented. Biologically, coordinating these genes is essential for establishing and maintaining trans-synaptic connections. Presynaptic organizers like *NRXN2* bind across the synaptic cleft to postsynaptic organizers such as *LRRTM1* to drive bidirectional synaptogenic signals. This trans-synaptic bridge subsequently relies on intracellular postsynaptic scaffolding proteins, including *DLGAP3* and *SHANK1*, to assemble the postsynaptic density, anchor neurotransmitter receptors, and ensure structural integrity for synaptic transmission [15]. However, our analysis revealed a prevalent transcriptional downregulation across these primary synaptic modules. This widespread deterioration aligns with established literature identifying synaptic loss and dysfunction as early, major correlates of cognitive decline in AD. Specifically, the expression deficits observed in the GABAergic and glutamatergic modules confirm the recognized excitatory/inhibitory imbalance in AD [16]. Furthermore, the severe suppression of neurotransmitter synthesis observed across these modules supports the validity and sensitivity of our bulk RNA-seq network approach in capturing the core pathological hallmarks of LOAD.

### 3.2. Quantifying the Suspected Network Collapse and Fragmentation

While recent theories have proposed that AD pathology is driven by an imbalance in cellular communication leading to a homeostatic disintegration of the network organization, our methodology provides concrete quantitative evidence of this structural fragmentation. While Pérez-González et al. (2025) [12] identified synaptic genes as globally enriched in betweenness centrality within the AD network, the present analysis extends this finding by resolving how specific genes within canonical pathways redistribute their topological position under pathological conditions, as quantified by quadrant redistribution and intra-pathway shortest-path distances, providing a pathway-resolved characterization that was not performed in the prior study. We demonstrated that the AD network undergoes a mesoscopic reorganization characterized by an increased modular segregation (Q = 0.28 vs. Q = 0.20) and rearrangement of gene membership (NMI = 0.4479). Biologically, this higher modularity score means that interactions between gene co-expression modules become more isolated from one another under disease conditions. This structural fragmentation is highly important because it leads to reduced cross-talk between different functional communities. Notably, edge-level analysis revealed that this increased modularity arises primarily from stronger internal cohesion within communities rather than a reduction in absolute inter-community connectivity, the AD network showed a higher proportion of inter-community edges (3.8%, *n* = 1058) compared to control (2.2%, *n* = 606), suggesting that co-expression becomes more compartmentalized under pathological conditions. Reflecting a disrupted regulatory coordination and a loss of efficient flow across the transcriptomic program, this ultimately points to a fundamental breakdown in cellular communication during AD progression [13]. A key example of this structural loosening is *GABRD*, which encodes the delta subunit of the GABA-A receptor. *GABRD* shifted from Quadrant III to Quadrant IV, demonstrating a marked loss of network connectivity. Since this subunit plays a critical role in providing a persistent, low-level inhibitory conductance that stabilizes neuronal excitability, its reduced connectivity quantitatively proves how the loss of this sustained inhibitory “brake” may contribute to the suspected neuronal hyperexcitability observed in AD [17]. It is important to distinguish, however, between two types of changes observed in this analysis. A reduction in expression levels would imply a direct loss of inhibitory function through decreased protein availability. In contrast, the reduction in transcriptional connectivity observed for GABRD suggests that while the inhibitory machinery may remain present, its coordinated regulation with other pathway components is disrupted, representing a loss of regulatory flexibility rather than an absolute loss of function. This distinction highlights the value of network-based approaches in capturing subtler forms of transcriptional dysregulation that expression-level analyses alone would not detect [7].

The biological consequences of this structural fragmentation are not merely topological abstractions. The increased intra-pathway shortest-path distances observed across all four canonical pathways suggest a progressive decoupling of functionally related synaptic genes, which may translate into impaired coordination of neurotransmitter release, synaptic vesicle cycling, and trans-synaptic signaling [18]. Specifically, the expansion of path distances within the Synaptogenesis and SNARE pathways is consistent with a disruption in the transcriptional co-regulation of genes required for synaptic vesicle fusion and membrane docking [19]. Moreover, the condition-specific enrichment patterns observed, with GABAergic signaling enriched exclusively in control and glutamatergic signaling exclusively in AD, suggest a pathological shift in the balance of inhibitory and excitatory transcriptional programs, providing a molecular correlate of the excitatory/inhibitory imbalance documented in AD neuropathology [20,21].

### 3.3. Evidence of Targeted Adaptation and Topological Centralization

The overall contribution of this study is the discovery of topological centralization via quadrant redistribution. Genes previously occupying lower connectivity states (Quadrant II), such as *GABRA1* and *GABRA2*, *SYN2*, *STX1B*, *SNCA* and *WASF1*, show a statistical redistribution toward higher co-expression centrality (Quadrant I) in the AD network. This pattern is consistent with the hypothesis that the AD transcriptome reorganizes co-expression around specific synaptic genes, though MI edges reflect statistical dependencies rather than physical interactions or directed signaling events. It is important to note that we interpret these findings strictly as disease-associated co-expression reorganization patterns. The present analysis does not claim that this topological rewiring acts as the primary causal factor driving disease progression, but rather a molecular signature of pathological state. Because this is a newly identified transcriptomic adaptation, future experimental validation will be necessary to contextualize how effectively these topological shifts translate to functional protein-level compensation.

### 3.4. Diverging from Traditional Degradation Models

Traditional differential expression analyses often portray AD as a uniform, passive degradation of synaptic cascades. Our integration of topology and expression contradicts this assumption, as discussed in Section 3.3. By applying condition-specific mean expression values alongside node degree, we observed that several genes gained topological influence in the disease state despite reduced or below-median expression levels. This discrepancy arises because traditional analyses rely on static, historically established signaling maps, whereas our data-driven co-expression framework captures the structural reorganization of the transcriptome under pathological conditions. Precisely because this reorganization is detectable at the pathway level, it offers a foundation for future hypothesis generation, though a detailed exploration of clinical or therapeutic applications falls outside the scope of the present work. The transcriptional co-expression patterns described here present a data-driven, in silico characterization of network reorganization in AD, and experimental validation of the functional relevance of these topological shifts remains a necessary prerequisite before any translational implications can be responsibly proposed. These findings are therefore best understood as a systems-level framework that may inform, but not prescribe, future research into targeted interventions.

### 3.5. Limitations

Despite the robust methodological approach, several limitations must be transparently acknowledged. First, the cross-sectional design of the ROSMAP [22] RNA-seq data restricts the ability to infer temporal trajectories or sequential rewiring events during the chronological progression of LOAD. Second, the use of bulk RNA-seq data introduces cell-type heterogeneity, which acts as a major uncontrolled confound. Bulk brain tissue aggregates and mixes expression signals from neurons, microglia, astrocytes, and oligodendrocytes. Because LOAD is characterized by shifts in cellular composition, such as severe neuronal loss, microglial activation, and reactive astrogliosis, the observed gene expression levels are heavily influenced by the proliferation or death of these specific cell types. Without applying computational deconvolution algorithms to mathematically separate the contributions of cell-type proportions, changes driven by cellular composition shifts are indistinguishable from genuine, cell-autonomous transcriptional rewiring [23]. Third, the local structural characterization utilized a pathway-focused shortest-path approach based on standardized ring structures constructed via igraph [24]; while informative for local efficiency, this method isolates pathways from the broader global interactome, potentially obscuring wider inter-pathway distances. Furthermore, the targeted evaluation of synaptic pathways was a post hoc selection. Although justified by statistical overrepresentation, this limits unbiased functional discovery outside of those specific neurobiological domains. Nevertheless, the four pathways selected, GABAergic signaling, SNARE complex, Synaptogenesis, and Neurexins and Neuroligins, are independently supported by extensive prior literature as core components of AD pathophysiology. GABAergic dysfunction, including downregulation of GABA-A and GABA-B receptors and excitatory/inhibitory imbalance, has been consistently documented in post-mortem AD brain tissue [21,25]. SNARE complex proteins are markedly reduced in AD brains, with Aβ oligomers directly blocking SNARE-mediated vesicle fusion through interaction with Syntaxin 1a [19]. Finally, synaptic loss is among the earliest and most robust correlates of cognitive decline in LOAD, preceding neuronal death [18]. Therefore, while the selection was statistically driven, it converges with well-established neurological evidence, strengthening the biological plausibility of our findings. Lastly, our functional interpretations rely heavily on curated knowledge bases (such as IPA), which are inherently constrained by the current limits of scientific literature and may be subject to annotation bias.

## 4. Materials and Methods

**Figure 11:**
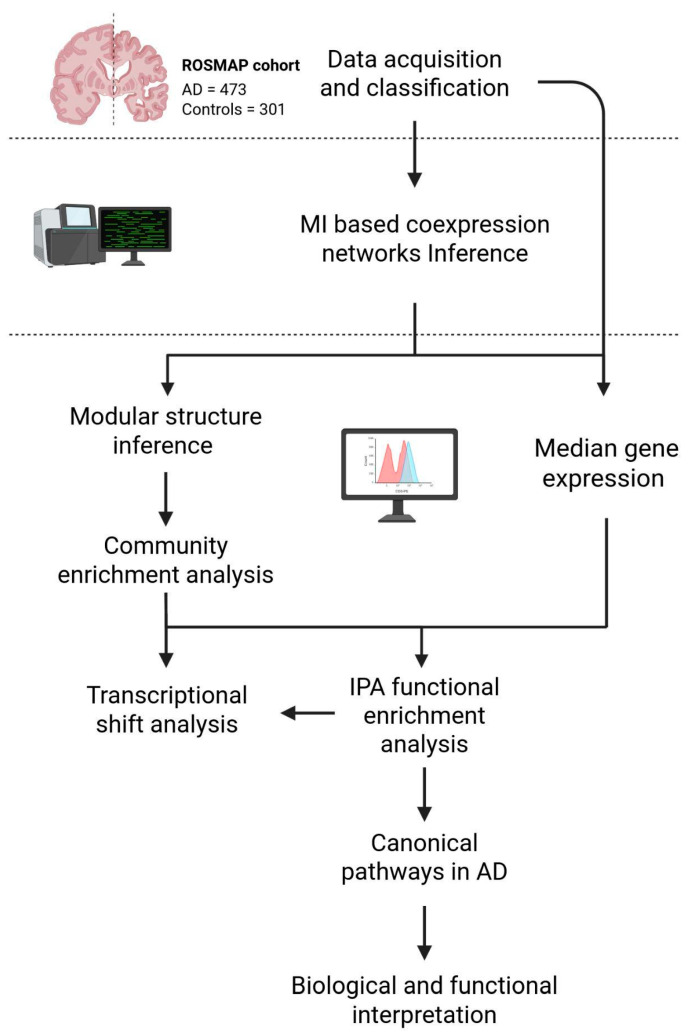
Workflow for Canonical Pathway Identification via Network Analysis in Alzheimer’s Disease. Dashed lines separate the three main methodological phases: (1) data acquisition and classification, (2) inference of co-expression networks, and (3) community enrichment and IPA functional analysis. Arrows indicate the pipeline’s sequential flow (illustrative images were created in BioRender.com) Adapted from Pérez-González et al. [12].

The methodology used in this study focused on analyzing gene co-expression networks to characterize the transcriptional structure and organization of LOAD in the DLPFC. The workflow was divided into the following stages: data acquisition, classification and quality control, inference of gene co-expression networks, and functional enrichment analysis using Ingenuity Pathway Analysis (IPA) [14] (see Figure 11).

Building upon previous systems-level findings, this study serves as a direct continuation of the network-based characterization of LOAD established by [12]. Accordingly, the conditions-specific gene co-expression networks analyzed herein were obtained from the foundational framework. Briefly, the networks were constructed using DLPFC bulk RNA-seq data from the ROSMAP cohort (473 AD and 301 control subjects). Raw counts were filtered (≥1 CPM), batch corrected with ARSyNSeq [26], and normalized using the Trimmed-Mean-of-M-values (TMM) method. The resulting expression matrices were stratified by NIA-Reagan [27] diagnosis based on metadata to create separate AD and control datasets. AD cases were selected considering both the intermediate and high likelihood categories as a positive diagnosis. Average expression and standard deviation values, corresponding to arithmetic summaries of normalized counts, were computed separately for each biological condition.

**Figure 12:**
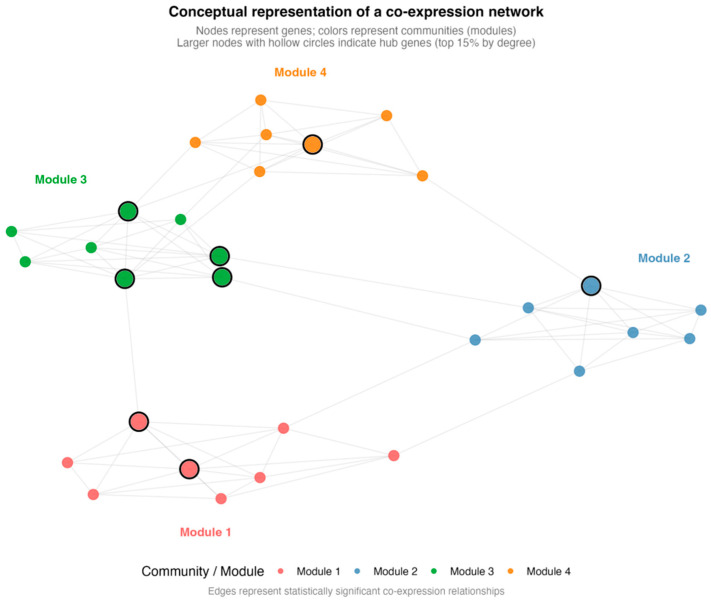
Conceptual representation of a gene co-expression network. Each node represents an individual gene and each edge represents a statistically significant co-expression relationship between two genes. Node colors indicate distinct communities (modules), defined as groups of genes more densely with community assignments. Larger nodes represent hub genes.

For each group, mutual information between gene expression was calculated using the Infotheo R package (v1.2.0.1) [28]. To retain only the strongest dependencies, a heuristic cut at the 99.99 percentile of MI values was applied. This specific threshold was selected following the systematic evaluation reported in Pérez-González et al. (2025) [12], where the network metrics, including modularity, mean degree, number of communities, and mean shortest-path distance, were assessed across percentiles ranging from 80% to 99.9999%. The 99.99th percentile was identified as the optimal threshold that filters noise while preserving the largest connected components and essential topological features. By restricting the network to these highest MI values, we ensure that only the interactions with the most robust dependencies are included, producing undirected networks with identical numbers of edges for fair comparison. Network topology was examined with the igraph package (v2.2.2). In this framework, each node represents a gene and each edge represents a strong statistical dependency in expression between two genes (see Figure 12 for a conceptual representation of network structure and community organization. Analysis focused on degree and betweenness centrality to identify hub and high betweenness genes. Degree values represent the number of co-expression connections a gene has within the network, reflecting its local connectivity. Community labels correspond to modules identified via infomap [29] partitioning. 

Community structure was inferred with the Infomap algorithm, yielding 68 AD modules and 71 control modules. To determine whether the transcriptional modules identified in the control network are preserved, partially reorganized or completely rewired in the AD network, community similarity between the independently inferred AD and control co-expression networks were quantified using the Jaccard index. To assess the statistical significance of the observed Jaccard similarity, a community label permutation test was performed. For each permutation, the community labels of the control network were randomly shuffled while preserving the number and size of communities, and the mean maximum Jaccard similarity was computed between the permuted control partition and the AD partition. This procedure was repeated 1000 times to generate a reference distribution of Jaccard similarities expected by chance. A two-tailed empirical *p*-value was calculated as the proportion of permuted similarities as extreme as the observed value. Z-scores and empirical 95% confidence intervals were derived following the same procedure described for the shortest distance permutation analysis. All permutation analyses were performed with a fixed random seed (set.seed(42) in R Studio using R version 4.5.1 (2025-06-13). Specifically, for each community identified in the control network, Jaccard similarity was computed based on gene membership overlap. Functional enrichment of modules and central nodes was performed using clusterProfiler on GO Biological Process terms (*p*-value < 0.05, False Discovery Rate [FDR] < 0.05).

Functional enrichment and pathway analysis were performed using Ingenuity Pathway Analysis (IPA) Qiagen (Hilden, Germany), a knowledge-based bioinformatics platform that maps gene lists with quantitative metrics onto curated molecular interaction databases to identify statistically over-represented biological pathways and functional processes. Two input tables were independently submitted to this platform, each corresponding to one biological condition (control and AD). These tables contained each gene metric derived from the network analyses, including: average expression standard deviation, node degree within the inferred co-expression network, and community assignment as determined by network partitioning.

Upon submission, IPA mapped gene identifiers to its proprietary knowledge base and performed enrichment analyses, integrating experimentally validated and literature-derived relationships encompassing canonical pathways, upstream regulators, molecular interactions, disease associations, and functional annotations. Enrichment statistics were computed internally using right-tailed Fisher’s exact tests to evaluate the overrepresentation of input genes within curated gene sets. To ensure robust statistical significance and correct for multiple comparisons, an FDR threshold of <0.05 was applied. This threshold thereby complemented the user-provided quantitative and network-derived metrics with knowledge-driven biological context, enabling integration of transcriptomics, network topology, and curated pathways knowledge to generate a multidimensional functional interpretation of specific network structure.

The structural reorganization of synaptic and metabolic pathways between AD and control conditions was evaluated by integrating expression magnitude with community overlap and graph derived distanced metrics.

Shortest-path distances were computed for pathway-specific gene sets corresponding to the canonical pathways in Alzheimer and control samples using the original co-expression networks. Gene identifiers from the selected pathway were first filtered, cleaned, and intersected with the node sets of each condition-specific graph to retain only the genes present in the respective networks. For each condition, an induced subgraph was then constructed from the full network, preserving the original topology among the selected genes rather than imposing an artificial structure. Pairwise shortest-path distances between all nodes within each subgraph were calculated using the distances() function from the igraph package, generating symmetric distance matrices representing the minimum number of edges separating each pair of genes. Infinite matrices, corresponding to disconnected node pairs, were excluded from summary statistics. Mean shortest-path distances were computed for each condition as a global measure of network proximity within the pathway. The resulting distance matrices were visualized as clustered heatmaps using hierarchical clustering and exported for analyses. To assess the statistical significance of the observed mean shortest-path distances, a label permutation test was performed for each canonical pathway and condition. For each permutation, a random set of genes of equal size to the pathway gene set was sampled from the full network, and the mean shortest-path distance was computed within the resulting induced subgraph. This procedure was repeated 1000 times to generate a reference distribution of mean distances representing what would be expected if the pathway gene set were composed of randomly selected genes. A two-tailed empirical *p*-value was calculated as the proportion of permuted distances as extreme as the observed value. Z-scores were computed as the standard deviations separating the observed mean from the null distribution mean. Empirical 95% confidence intervals were derived from the 2.5th and 97.5th percentiles of the null distributions. All permutation analyses were performed with a fixed random seed (set.seed(42) in R Studio) to ensure reproducibility.

In conclusion, our multidimensional network analysis reveals that LOAD is not merely a disease of isolated transcriptional deficits, but complex, active structural rewiring of the brain’s functional architecture. The AD transcriptome shows a co-expression pattern consistent with a shift from integrated modular organization to increased segregation, with specific hub genes displaying statistically elevated centrality within synaptic pathways. Identifying these highly centralized genes represents a statistically grounded starting point for hypothesis generation, and future experimental validation will be necessary to determine whether these co-expression patterns reflect functionally relevant targets for the therapeutic intervention in AD.

## Figures and Tables

**Table 1:** Operational definition of the median defined quadrants representing the shifts in gene interconnectivity and expression, relative to the threshold boundaries.

**Quadrant II**	**Quadrant I**
Low interconnectivity degree, high average expression	High interconnectivity degree, high average expression
**Quadrant IV**	**Quadrant III**
Low interconnectivity degree, low average expression	High interconnectivity degree, low average expression

**Table 2:** Comparison of mean distances across canonical pathways between the Control and AD groups.

Pathway	Control Mean Distance	AD Mean Distance
GABAergic	2.293	2.435
Neurexins and neuroligins	1.571	1.846
SNARE	2.183	2.765
Synaptogenesis	1.894	2.708

## Data Availability

ROSMAP data can be requested at http://www.radc.rush.edu (accessed on 20 May 2026). All code involved in this work is publicly available at the following link: https://github.com/CSB-IG/ROSMAP_MultiOmics/tree/main/ROSMAP-rnaseq-networks (accessed on 3 March 2025).

## Supplementary Materials

The following supporting information can be downloaded at https://www.mdpi.com/article/10.3390/ijms27114835/s1.
