# Supplementary figures and images for: Canonical Pathways Rewiring in Alzheimer’s Disease
Source: Int J Mol Sci. 2026 May 27;27(11):4835. doi: 10.3390/ijms27114835 (PMC13256824; doi:10.3390/ijms27114835)

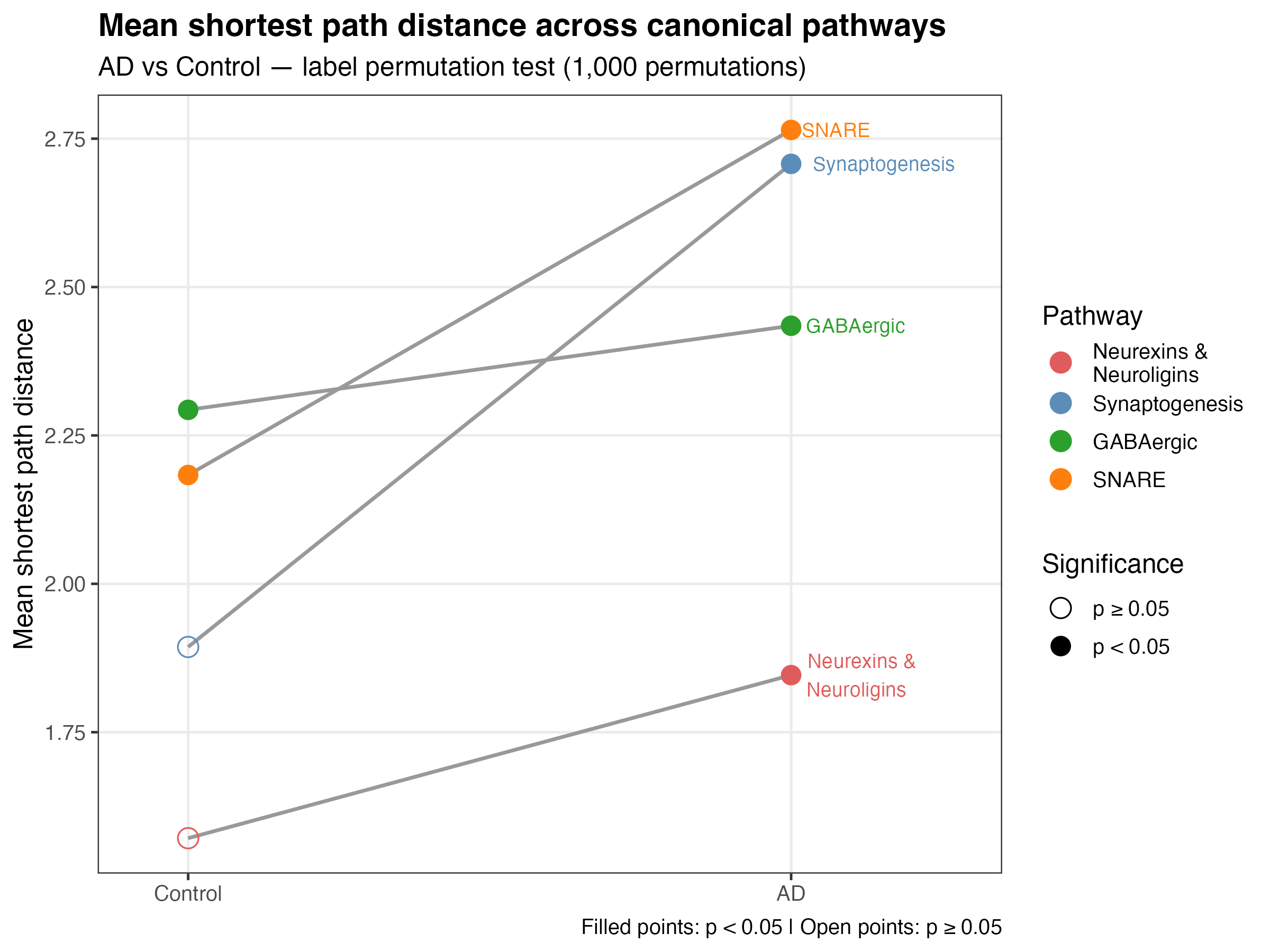

Supplement: Supplementary file 1 [file ijms-27-04835-s001.zip › ijms-4289742-supplementary/Figure_S1.png]
